# Supplementary material for: Dietary Diversity and Nutritional Adequacy among an Older Spanish Population with Metabolic Syndrome in the PREDIMED-Plus Study: A Cross-Sectional Analysis
Source: Nutrients. 2019 Apr 26;11(5):958. doi: 10.3390/nu11050958 (PMC6567048; doi:10.3390/nu11050958)
Supplement: Supplementary file 1 [file nutrients-11-00958-s001.zip › Supplementary Table 1_TrackedCopy.docx]

**Table S1.** Percentage of participants of PREDIMED-Plus study with nutrient intake below 2/3 of DRIs according to DDS.

| **Nutrient** | **Group** | **DRI^a^** | **Q1**  **(n=1647)** | **Q2**  **(n=1647)** | **Q3 (n=1647)** | **Q4**  **(n=1646)** | **P value^1^** |
| --- | --- | --- | --- | --- | --- | --- | --- |
| **Dietary fiber** | Male 55-70 | 30g /d | 49.6 | 25.9 | 12.0 | 4.7 | <0.001 |
|  | Male >70 | 30g /d | 38.8 | 32.1 | 12.9 | 5.5 | <0.001 |
|  | Female 60-70 | 21 g/d | 13.4 | 4.6 | 1.2 | 0.4 | <0.001 |
|  | Female >70 | 21 g/d | 18.0 | 5.7 | 0.7 | 0 | <0.001 |
| **P value^2^** |  |  | <0.001 | <0.001 | <0.001 | <0.001 |  |
| **Vitamin A** | Male 55-70 | 900 µg/d | 33.8 | 15.7 | 12.0 | 3.5 | <0.001 |
|  | Male >70 | 900 µg/d | 31.9 | 20.2 | 9.9 | 7.7 | <0.001 |
|  | Female 60-70 | 700 µg/d | 14.9 | 8.4 | 3.4 | 0.7 | <0.001 |
|  | Female >70 | 700 µg/d | 22.5 | 12.3 | 6.3 | 0 | <0.001 |
| **P value^2^** |  |  | <0.001 | <0.001 | <0.001 | <0.001 |  |
| **Vitamin B_9_** | Male 55-70 | 400 µg/d | 40.6 | 15.7 | 8.8 | 3.0 | <0.001 |
|  | Male >70 | 400 µg/d | 33.6 | 25.7 | 6.9 | 5.5 | <0.001 |
|  | Female 60-70 | 400 µg/d | 39.4 | 24.8 | 13.5 | 3.1 | <0.001 |
|  | Female >70 | 400 µg/d | 46.1 | 27.9 | 12.6 | 5.0 | <0.001 |
| **P value^2^** |  |  | 0.31 | <0.001 | 0.020 | 0.33 |  |
| **Vitamin D** | Male 60-70 | 15 µg/d | 88.5 | 84.0 | 80.1 | 72.5 | <0.001 |
|  | Male >70 | 20 µg/d | 98.3 | 99.1 | 97.0 | 95.6 | 0.401 |
|  | Female 60-70 | 15 µg/d | 89.1 | 84.2 | 82.3 | 76.2 | <0.001 |
|  | Female >70 | 20 µg/d | 96.6 | 98.4 | 98.6 | 97.0 | 0.66 |
| **P value^2^** |  |  | 0.001 | <0.001 | <0.001 | <0.001 |  |
| **Vitamin E** | Male 55-70 | 15 mg/d | 61.7 | 46.6 | 42.5 | 29.3 | <0.001 |
|  | Male >70 | 15 mg/d | 62.1 | 56.0 | 43.6 | 30.8 | <0.001 |
|  | Female 60-70 | 15 mg/d | 67.2 | 57.8 | 53.1 | 35.3 | <0.001 |
|  | Female >70 | 15 mg/d | 79.8 | 69.7 | 59.4 | 50.3 | <0.001 |
| **P value^2^** |  |  | 0.003 | <0.001 | <0.001 | <0.001 |  |
| **Calcium** | Male 55-70 | 1000 mg/d | 24.4 | 9.4 | 4.8 | 0.1 | <0.001 |
|  | Male >70 | 1200 mg/d | 48.3 | 26.6 | 18.8 | 9.9 | <0.001 |
|  | Female 60-70 | 1200 mg/d | 49.1 | 35.6 | 20.7 | 7.1 | <0.001 |
|  | Female >70 | 1200 mg/d | 41.6 | 36.1 | 18.9 | 10.6 | <0.001 |
| **P value^2^** |  |  | <0.001 | <0.001 | <0.001 | <0.001 |  |
| **Magnesium** | Male 55-70 | 420 mg/d | 14.6 | 4.1 | 1.8 | 0.4 | <0.001 |
|  | Male >70 | 420 mg/d | 15.5 | 6.4 | 2.0 | 1.1 | <0.001 |
|  | Female 60-70 | 320 mg/d | 3.2 | 1.2 | 0.3 | 0 | <0.001 |
|  | Female >70 | 320 mg/d | 3.4 | 0.8 | 0 | 0 | 0.011 |
| **P value^2^** |  |  | <0.001 | 0.001 | 0.012 | 0.06 |  |
| **Iodine** | Male 55-70 | 150 µg/d | 17.7 | 9.4 | 7.6 | 3.3 | <0.001 |
|  | Male >70 | 150 µg/d | 24.1 | 11.0 | 9.9 | 3.3 | <0.001 |
|  | Female 60-70 | 150 µg/d | 22.6 | 14.5 | 8.3 | 4.5 | <0.001 |
|  | Female >70 | 150 µg/d | 13.5 | 9.8 | 6.3 | 3.0 | 0.007 |
| **P value^2^** |  |  | 0.037 | 0.029 | 0.73 | 0.63 |  |

DRI^a^: Dietary Reference Intake. Pearson´s Chi-Square test was used to estimate differences among prevalence of inadequate nutrient intakes according to quartiles of DDS for each age and sex strata (*p value^1^*) and also to estimate differences among prevalence of inadequate nutrient intakes according to age and sex, for each DDS quartile (*p value^2^*).

Abbreviations: DDS, dietary diversity score; DRI, dietary reference intake.
