# Supplementary material for: Dietary Diversity and Nutritional Adequacy among an Older Spanish Population with Metabolic Syndrome in the PREDIMED-Plus Study: A Cross-Sectional Analysis
Source: Nutrients. 2019 Apr 26;11(5):958. doi: 10.3390/nu11050958 (PMC6567048; doi:10.3390/nu11050958)
Supplement: Supplementary file 1 [file nutrients-11-00958-s001.zip › Supplementary Table 2_Trackedcopy.docx]

**Table S2.** Percentage of participants in the PREDIMED-Plus study with nutrient intake below 2/3 of DRIs according to DDS

| **Nutrient** | **Group** | **DRI^a^** | **Q1**  **(n=1647)** | **Q2**  **(n=1647)** | **Q3 (n=1647)** | **Q4**  **(n=1646)** | **P value^1^** |
| --- | --- | --- | --- | --- | --- | --- | --- |
| **Vitamin B_1_** | Male 55-70 | 1.2 mg/d | 1.3 | 0 | 0 | 0 | <0.001 |
|  | Male >70 | 1.2 mg/d | 2.6 | 0 | 0 | 0 | 0.049 |
|  | Female 60-70 | 1.1 mg/ | 2.3 | 0 | 0 | 0 | <0.001 |
|  | Female >70 | 1.1 mg/d | 2.3 | 0.82 | 0 | 0 | <0.001 |
| **P value^2^** |  |  | 0.48 | 0.006 | - | - |  |
| **Vitamin B_6_** | Male 60-70 | 1.7 mg/d | 1.9 | 0 | 0 | 0 | <0.001 |
|  | Male >70 | 1.7 mg/d | 3.5 | 0 | 0 | 0 | 0.015 |
|  | Female 60-70 | 1.5 mg/d | 2.3 | 0.2 | 0 | 0 | <0.001 |
|  | Female >70 | 1.5 mg/d | 3.4 | 0.8 | 0 | 0 | 0.011 |
| **P value^2^** |  |  | 0.61 | 0.11 | - | - |  |
| **Vitamin B^12^** | Male 60-70 | 2.4 µg/d | 0.2 | 0 | 0 | 0 | 0.26 |
|  | Male >70 | 2.4 µg/d | 0 | 0 | 0 | 0 | - |
|  | Female 60-70 | 2.4 µg/d | 0.2 | 0 | 0 | 0 | 0.18 |
|  | Female >70 | 2.4 µg/d | 0 | 0.8 | 0 | 0 | 0.32 |
| **P value^2^** |  |  | 0.93 | 0.006 | - | - |  |
| **Vitamin C** | Male 60-70 | 90 mg/d | 3.7 | 0 | 0 | 0 | <0.001 |
|  | Male >70 | 90 mg/d | 5.2 | 0 | 0 | 0 | 0.001 |
|  | Female 60-70 | 75 mg/d | 1.8 | 0 | 0 | 0 | <0.001 |
|  | Female >70 | 75 mg/d | 1.1 | 0 | 0 | 0 | 0.16 |
| **P value^2^** |  |  | 0.09 | - | - | - |  |
| **Phosphorus** | Male 55-70 | 700 mg/d | 0 | 0 | 0 | 0 | - |
|  | Male >70 | 700 mg/d | 0 | 0 | 0 | 0 | - |
|  | Female 60-70 | 700 mg/d | 0.2 | 0 | 0 | 0 | 0.18 |
|  | Female >70 | 700 mg/d | 0 | 0 | 0 | 0 | - |
| **P value^2^** |  |  | 0.44 | - | - | - |  |
| **Iron** | Male 55-70 | 8 mg /d | 0 | 0 | 0 | 0 | - |
|  | Male >70 | 8 mg /d | 0 | 0 | 0 | 0 | - |
|  | Female 60-70 | 8 mg /d | 0.2 | 0 | 0 | 0 | 0.18 |
|  | Female >70 | 8 mg /d | 0 | 0 | 0 | 0 | - |
| **P value^2^** |  |  | 0.44 | - | - | - |  |
| **Potassium** | Male 55-70 | 4 g/d | 0 | 0 | 0 | 0 | - |
|  | Male >70 | 4 g/d | 0 | 0 | 0 | 0 | - |
|  | Female 60-70 | 4 g/d | 0 | 0 | 0 | 0 | - |
|  | Female >70 | 4 g/d | 0 | 0 | 0 | 0 | - |
| **P value^2^** |  |  | - | - | - | - |  |
| **Selenium** | Male 60-70 | 55 µg/d | 0.2 | 0 | 0 | 0 | 0.26 |
|  | Male >70 | 55 µg/d | 0 | 0 | 0 | 0 | - |
|  | Female 60-70 | 55 µg/d | 1.1 | 0.8 | 0 | 0.4 | <0.001 |
|  | Female >70 | 55 µg/d | 1.1 | 0 | 0 | 0 | 0.34 |
| **P value^2^** |  |  | 0.08 | 0.006 | - | - |  |
| **Zinc** | Male 55-70 | 11 mg/d | 3.3 | 0.7 | 0.8 | 0.2 | <0.001 |
|  | Male >70 | 11 mg/d | 2.6 | 0.9 | 0 | 0 | 0.17 |
|  | Female 60-70 | 8 mg/d | 1.1 | 0 | 0 | 0 | <0.001 |
|  | Female >70 | 8 mg/d | 2.3 | 0 | 0 | 0 | 0.015 |
| **P value^2^** |  |  | 0.13 | 0.13 | 0.06 | 0.53 |  |

DRI^a^: Dietary Reference Intake. Pearson´s Chi-Square test was used to estimate differences among prevalence of inadequate nutrient intakes according to quartiles of DDS for each age and sex strata (*p value^1^*) and also to estimate differences among prevalence of inadequate nutrient intakes according to age and sex, for each DDS quartile (*p value^2^*).

Abbreviations: DDS, dietary diversity score; DRI, dietary reference intake; Q, quartile.
