# Supplementary material for: Dietary Diversity and Nutritional Adequacy among an Older Spanish Population with Metabolic Syndrome in the PREDIMED-Plus Study: A Cross-Sectional Analysis
Source: Nutrients. 2019 Apr 26;11(5):958. doi: 10.3390/nu11050958 (PMC6567048; doi:10.3390/nu11050958)
Supplement: Supplementary file 1 [file nutrients-11-00958-s001.zip › Supplementary Table 3_TrackedCopy.docx]

**Table S3.** Participants of PREDIMED-Plus study with nutrient intake below AR/AI proposed by EFSA according to DDS

| **Nutrient** | **Group** | **AR/AI^a^** | **Q1**  **(n=1647)** | **Q2**  **(n=1647)** | **Q3 (n=1647)** | **Q4**  **(n=1646)** | **P value^1^** |
| --- | --- | --- | --- | --- | --- | --- | --- |
| **Dietary fiber** | Male | 25g /d | 74.8 | 58.5 | 43.4 | 24.7 | <0.001 |
|  | Female | 25 g/d | 72.1 | 59.2 | 46.5 | 25.5 | <0.001 |
| **P value^2^** |  |  | 0.25 | 0.77 | 0.20 | 0.70 |  |
| **Vitamin A** | Male | 570 µg/d | 30.6 | 12.9 | 9.6 | 3.0 | <0.001 |
|  | Female | 490 µg/d | 19.8 | 11.1 | 5.0 | 0.7 | <0.001 |
| **P value^2^** |  |  | <0.001 | 0.27 | <0.001 | <0.001 |  |
| **Vitamin B_1_** | Male | 0.072 mg/d | 0 | 0 | 0 | 0 | - |
|  | Female | 0.072 mg/d | 0 | 0 | 0 | 0 | - |
| **P value^2^** |  |  | - | - | - | - |  |
| **Vitamin B_6_** | Male | 1.5 mg/d | 13.9 | 3.0 | 1.8 | 0.7 | <0.001 |
|  | Female | 1.3 mg/d | 8.9 | 2.3 | 0.2 | 0 | <0.001 |
| **P value^2^** |  |  | 0.004 | 0.44 | 0.001 | 0.008 |  |
| **Vitamin B_9_** | Male | 250 µg/d | 31.6 | 11.9 | 5.5 | 1.8 | <0.001 |
|  | Female | 250 µg/d | 34.7 | 19.0 | 8.9 | 1.9 | <0.001 |
| **P value^2^** |  |  | 0.22 | <0.001 | 0.007 | 0.91 |  |
| **Vitamin B^12^** | Male | 4 µg/d | 4.8 | 2.2 | 2.0 | 0.3 | <0.001 |
|  | Female | 4 µg/d | 9.0 | 3.7 | 3.0 | 0.7 | <0.001 |
| **P value^2^** |  |  | 0.001 | 0.07 | 0.19 | 0.37 |  |
| **Vitamin C** | Male | 90 mg/d | 19.0 | 3.8 | 0.7 | 0.2 | <0.001 |
|  | Female | 80 mg/d | 11.1 | 1.4 | 0.2 | 0 | <0.001 |
| **P value^2^** |  |  | <0.001 | 0.002 | 0.19 | 0.19 |  |
| **Vitamin D** | Male | 15 µg/d | 99.5 | 98.8 | 98.3 | 97.3 | 0.003 |
|  | Female | 15 µg/d | 98.7 | 99.0 | 99.3 | 98.3 | 0.19 |
| **P value^2^** |  |  | 0.09 | 0.64 | 0.06 | 0.19 |  |
| **Vitamin E** | Male | 13 mg/d | 85.4 | 78.7 | 74.2 | 65.0 | <0.001 |
|  | Female | 11 mg/d | 79.1 | 70.6 | 68.2 | 52.9 | <0.001 |
| **P value^2^** |  |  | 0.001 | <0.001 | 0.008 | <0.001 |  |
| **Calcium** | Male | 950 mg/d | 64.6 | 45.7 | 36.9 | 19.0 | <0.001 |
|  | Female | 950 mg/d | 65.9 | 55.5 | 43.0 | 25.1 | <0.001 |
| **P value^2^** |  |  | 0.60 | <0.001 | 0.012 | 0.005 |  |
| **Phosphorus** | Male | 550 mg/d | 0.1 | 0 | 0 | 0 | 0.56 |
|  | Female | 550 mg/d | 0.2 | 0 | 0 | 0 | 0.17 |
| **P value^2^** |  |  | 0.591 | - | - | - |  |
| **Magnesium** | Male | 350 mg/d | 43.2 | 27.4 | 16.5 | 6.5 | <0.001 |
|  | Female | 300 mg/d | 29.2 | 17.1 | 10.0 | 1.9 | <0.001 |
| **P value^2^** |  |  | <0.001 | <0.001 | <0.001 | <0.001 |  |

AR/AI^a^: Average Requirements/Adequate intake according to EFSA criteria. DDS: dietary diversity score. EFSA: European Food Safety Authority. Q: Quartile.

Pearson´s Chi-Square test was used to estimate differences among prevalence of inadequate nutrient intakes according to quartiles of DDS for each age and sex strata (*p value^1^*) and also to estimate differences among prevalence of inadequate nutrient intakes according to age and sex, for each DDS quartile (*p value^2^*).

**Supplementary Table 3.** (Continued). Participants of PREDIMED-Plus study with nutrient intake below AR/AI proposed by EFSA according to DDS

| **Nutrient** | **Group** | **DRI^a^** | **Q1**  **(n=1647)** | **Q2 (n=1647)** | **Q3**  **(n=1647)** | **Q4**  **(n=1646)** | **P value^1^** |
| --- | --- | --- | --- | --- | --- | --- | --- |
| **Iron** | Male | 6 mg /d | 0.3 | 0 | 0 | 0 | 0.11 |
|  | Female | 6 mg /d | 0.6 | 0 | 0 | 0 | 0.002 |
| **P value^2^** |  |  | 0.35 | - | - | - |  |
| **Iodine** | Male | 150 µg/d | 34.2 | 19.7 | 16.8 | 10.2 | <0.001 |
|  | Female | 150 µg/d | 33.2 | 23.3 | 15.2 | 8.4 | <0.001 |
| **P value^2^** |  |  | 0.66 | 0.08 | 0.38 | 0.23 |  |
| **Potassium** | Male | 3.5 g/d | 0 | 0 | 0 | 0 | - |
|  | Female | 3.5 g/d | 0 | 0 | 0 | 0 | - |
| **P value^2^** |  |  | - | - | - | - |  |
| **Selenium** | Male | 70 µg/d | 9.4 | 4.9 | 3.1 | 2.0 | <0.001 |
|  | Female | 70 µg/d | 14.3 | 7.9 | 6.0 | 1.9 | <0.001 |
| **P value^2^** |  |  | 0.003 | 0.012 | 0.005 | 0.90 |  |
| **Zinc** | Male | 11 mg/d | 31.3 | 22.4 | 19.4 | 10.8 | <0.001 |
|  | Female | 8.9 mg/d | 20.0 | 11.8 | 7.3 | 2.9 | <0.001 |
| **P value^2^** |  |  | <0.001 | <0.001 | <0.001 | <0.001 |  |

AR/AI^a^: Average Requirements/Adequate intake according to EFSA criteria. Pearson´s Chi-Square test was used to estimate differences among prevalence of inadequate nutrient intakes according to quartiles of DDS for each age and sex strata (*p value^1^*) and also to estimate differences among prevalence of inadequate nutrient intakes according to age and sex, for each DDS quartile (*p value^2^*).
