# Supplementary material for: Dietary Diversity and Nutritional Adequacy among an Older Spanish Population with Metabolic Syndrome in the PREDIMED-Plus Study: A Cross-Sectional Analysis
Source: Nutrients. 2019 Apr 26;11(5):958. doi: 10.3390/nu11050958 (PMC6567048; doi:10.3390/nu11050958)
Supplement: Supplementary file 1 [file nutrients-11-00958-s001.zip › Supplementary Table 4_Trackedcopy.docx]

**Table S4. Number of inadequacies and** distribution of participants with ≥4 nutrients below EFSA criteria according to DDS stratified by sex

|  | **MEN** | | | | |
| --- | --- | --- | --- | --- | --- |
|  | **Q1 (n=787)** | **Q2 (n=763)** | **Q3 (n=973)** | **Q4 (n=489)** | **P value** |
| **Inadequacies, mean ±SD** | 3.5±0.8 | 3.1±1.0 | 2.8±1.1 | 2.2±1.0 | <0.001^1^ |
| **Participants, n (%)** | 778 (69.7) | 432 (47.2) | 253 (32.9) | 81 (13.5) | <0.001^2^ |
|  | **WOMEN** | | | | |
|  | **Q1 (n=630)** | **Q2 (n=610)** | **Q3 (n=884)** | **Q4 (n=529)** | **P value** |
| **Inadequacies, mean (SD)** | 3.4±0.8 | 3.1±1.0 | 2.7±1.1 | 2.1±1.1 | <0.001^1^ |
| **Participants, n (%)** | 335 (63.1) | 330 (45.1) | 284 (32.4) | 136 (13.0) | <0.001^2^ |

^1^P value: Pearson´s Chi-Square test was used to estimate differences among prevalence of inadequate nutrient intake according to quartiles of DDS for sex strata. ^2^P value: ANOVA test was performed to estimate differences among mean of inadequate nutrient intakes according for each DDS quartile**.**

Abbreviations: AR/AI, Average Requirements/Adequate intake according to EFSA criteria; DDS, dietary diversity score; EFSA, European Food Safety Authority; Q, quartile.
