# Supplementary material for: Dietary Diversity and Nutritional Adequacy among an Older Spanish Population with Metabolic Syndrome in the PREDIMED-Plus Study: A Cross-Sectional Analysis
Source: Nutrients. 2019 Apr 26;11(5):958. doi: 10.3390/nu11050958 (PMC6567048; doi:10.3390/nu11050958)
Supplement: Supplementary file 1 [file nutrients-11-00958-s001.zip › Supplementary Table 5_Trackedcopy.docx]

**Table S5.** Multivariable logistic regression models for inadequate intake of 4 or more out 8 micronutrients according to EFSA AR/AI by food group’s diversity intake and total DDS quartiles in the PREDIMED-Plus study participants. Odds ratios (95% Confidence intervals).

|  | **Q1**  **(n=1647)** | **Q2**  **(n=1647)** | **Q3**  **(n=1647)** | **Q4**  **(n=1646)** |
| --- | --- | --- | --- | --- |
| **Total DDS** | | | | |
| Model 1 | 13.73 (11.51-16.37) | 5.67 (4.77-6.74) | 3.19 (2.67-3.80) | 1 (Ref.) |
| Model 2 | 14.48 (11.93-17.57) | 5.50 (4.57-6.60) | 3.17 (2.64-3.82) | 1 (Ref.) |
|  | **C1**  **(n=550)** | **C2**  **(n=1315)** | **C3**  **(n=2482)** | **C4**  **(n=2240)** |
| **Vegetable food group** | | | | |
| Model 1 | 10.73 (8.41-13.71) | 5.47 (4.62-6.48) | 2.34 (2.03-2.70) | 1 (Ref.) |
| Model 2 | 8.11 (6.13-10.73) | 4.90 (4.01-5.93) | 2.15 (1.83-2.53) | 1 (Ref.) |
|  | **C1**  **(n=845)** | **C2**  **(n=4497)** | **C3**  **(n=779)** | **C4**  **(n=466)** |
| **Fruit food group** | | | | |
| Model 1 | 12.31 (8.89-17.01) | 2.88 (2.17-3.84) | 1.68 (1.21-2.33) | 1 (Ref.) |
| Model 2 | 6.66 (4.61-9.62) | 2.05 (1.49-2.84) | 1.45 (1.00-2.10) | 1 (Ref.) |
|  | **C1**  **(n=350)** | **C2**  **(n=4767)** | **C3**  **(n=1390)** | **C4**  **(n=80)** |
| **Cereal food group** |  |  |  |  |
| Model 1 | 1.30 (0.68-2.52) | 1.13 (0.61-2.07) | 1.07 (0.57-1.98) | 1 (Ref.) |
| Model 2 | 0.71 (0.34-1.49) | 0.74 (0.37-1.47) | 0.77 (0.38-1.55) | 1 (Ref.) |
|  | **C1**  **(n=26)** | **C2**  **(n=1254)** | **C3**  **(n=2770)** | **C4**  **(n=2537)** |
| **Protein food group** |  |  |  |  |
| Model 1 | 7.19 (1.94-26.67) | 3.00 (2.56-3.53) | 1.89 (1.66-2.15) | 1 (Ref.) |
| Model 2 | 3.91 (0.77-19.72) | 1.93 (1.61-2.32) | 1.48 (1.28-1.72) | 1 (Ref.) |
|  | **C1**  **(n=686)** | **C2**  **(n=2447)** | **C3**  **(n=2600)** | **C4**  **(n=854)** |
| **Dairy food group** |  |  |  |  |
| Model 1 | 23.13 (17.18-31.13) | 6.61 (5.23-8.36) | 2.67 (2.11-3.38) | 1 (Ref.) |
| Model 2 | 17.88 (12.84-24.90) | 5.14 (3.96-6.67) | 2.19 (1.69-2.85) | 1 (Ref.) |
|  |  |  |  |  |
|  | | | | |
|  |  |  |  |  |
|  |  |  |  |  |

Model 1: Adjusted for energy intake^.^ Model 2: Adjusted for energy intake, sex, age, smoking habits, physical activity, educational level, MedDiet adherence, BMI, WC, alcohol intake, living alone and civil status.

Abbreviations: AR/AI, Average Requirements/Adequate intake according to EFSA criteria; C, category; DDS, dietary diversity score; EFSA, European Food Safety Authority; Q, quartile.
